# Peste Des Petits Ruminants in Pakistan: Current Status, Challenges and Prospects for Vaccine Development

**DOI:** 10.3390/vaccines13111101

**Published:** 2025-10-28

**Authors:** Abdul Kabir, Asghar Ali Kamboh, Muhammad Abubakar, Aness Ur Rahman, Muhammad Tayyab Jabbar, Muhammad Shafiq, Meirui Lin

**Affiliations:** 1 Department of Veterinary Microbiology, Faculty of Animal Husbandry and Veterinary Sciences, Sindh Agriculture University, Tando Jam 70060, Pakistan; kabirvet32@gmail.com (A.K.); drasgharkamboh@yahoo.com (A.A.K.); 2 National Veterinary Laboratory, Park Road, Islamabad 45500, Pakistan; mabnvl@gmail.com; 3 Department of Veterinary Medicine, Faculty of Animal Husbandry and Veterinary Sciences, Sindh Agriculture University, Tando Jam 70060, Pakistan; aneessafi1@gmail.com; 4 Department of Microbiology, Faculty of Veterinary and Animal Sciences, The Islamic University of Bahawalpur, Bahawalpur 63100, Pakistan; tayyabjabbar157@gmail.com; 5 Department of Pharmacology, Section Clinical Pharmacy, Shantou University Medical College, Shantou 515041, China; 6 Intensive Care Unit, First Affiliated Hospital of Shantou University Medical College, Shantou 515041, China

**Keywords:** peste des petits ruminants, Pakistan, epidemiology, eradication strategy, vaccines, thermostable formulations, novel delivery methods

## Abstract

Peste des petits ruminants (PPR) is a highly contagious viral disease that affects goats and sheep, causing severe clinical signs, high mortality, and significant economic losses in many developing countries. Pakistan is one of the endemic regions where PPR outbreaks caused by the Asian lineage IV virus have been reported frequently, affecting the livelihoods of millions of smallholder farmers who depend on these animals for food security and income generation. In this review, we provide a comprehensive overview of the current status, challenges and prospects for vaccine development against PPR in Pakistan. We discuss the epidemiology, diagnosis, and control of PPR in Pakistan, as well as the existing vaccines based on the attenuated strains and their limitations, such as low thermostability, short shelf life, and inability to differentiate between infected and vaccinated animals. We also highlight the recent advances in vaccine research and development, such as recombinant and vectored vaccines, thermostable formulations, and novel delivery methods that could overcome these limitations and enhance the immunogenicity and safety of PPR vaccines. We review the current and potential strategies for vaccine deployment, such as mass vaccination, targeted vaccination, ring vaccination, and their implications for the global eradication of PPR by 2030. We conclude by providing some recommendations for future research and development to improve vaccine efficacy, safety, and coverage in Pakistan, as well as to monitor the impact of vaccination on PPR incidence and prevalence.

## 1. Introduction

Peste des petits ruminants (PPR) is a highly contagious viral disease that affects goats and sheep, causing severe clinical signs, high mortality, and significant economic losses in many developing countries [1]. PPR is caused by PPR virus (PPRV), a member of the genus Morbillivirus and the family Paramyxoviridae, which is closely related to rinderpest virus (RPV), measles virus (MV), and canine distemper virus (CDV) [2,3]. PPRV has a single-stranded, negative-sense RNA genome of approximately 16 kb, encoding six structural proteins (nucleoprotein, phosphoprotein, matrix protein, fusion protein, hemagglutinin protein, and large polymerase protein) and two non-structural proteins (C and V) [4,5,6,7,8]. While Peste des petits ruminants virus (PPRV) has historically been confined to Asia and Africa, recent incursions into parts of Europe, including Bulgaria and Georgia, underscore its transboundary potential. The virus is classified into four lineages (I-IV) based on the fusion (F) gene. Among these, lineage IV is the most prevalent and is responsible for its widespread distribution across Africa, the Middle East, Asia, and its recent spread into Europe [9,10,11]. The disease is transmitted mainly through direct contact with infected animals or their secretions and excretions, such as saliva, nasal discharge, feces, and urine [12,13]. The incubation period ranges from 2 to 10 days, followed by the onset of fever, depression, anorexia, ocular and nasal discharge, erosive stomatitis, pneumonia, diarrhea, and dehydration [14,15,16]. The morbidity and mortality rates vary depending on the host species, breed, immune status, virus strain, and environmental factors, but can reach up to 90% and 80%, respectively [17].

PPR has a significant impact on the livelihoods of millions of smallholder farmers who depend on small ruminants for food security and income generation. PPR also poses a threat to wildlife conservation, as several wild ungulate species are susceptible to PPRV infection and may act as reservoirs or spillover hosts [18]. The global control and eradication of PPR is a priority for the international community, as part of the One Health initiative, which recognizes the interconnection between human, animal, and environmental health [19]. In 2015, the Food and Agriculture Organization of the United Nations (FAO) and the World Organization for Animal Health (OIE) launched the Global Strategy for the Control and Eradication of PPR, with the goal of eradicating PPR by 2030 [20]. The strategy is based on four stages: assessment, control, eradication, and post-eradication. The main tools for PPR control and eradication are surveillance, diagnosis, and vaccination. Vaccination is the most effective and cost-effective intervention to prevent and control PPR outbreaks. Currently, there are several live attenuated vaccines available against PPR, based on different strains of PPRV, such as Nigeria 75/1, Sungri 96, Arriah, Egypt 87, Ivory Coast 89, and Arasur 87 [21,22,23]. These vaccines are safe, immunogenic, and cross-protective against different lineages of PPRV [9]. However, they also have some limitations, such as the need for a cold chain, the inability to differentiate between infected and vaccinated animals (DIVA), the potential risk of reversion to virulence, and the interference with maternal antibodies [24]. Therefore, there is a need for the development of new generation vaccines against PPR that can overcome these limitations and enhance the global eradication efforts. In this review, we provide an overview of the current status, challenges, and prospects for vaccine development against PPR in Pakistan. We discuss the epidemiology, diagnosis, and control of PPR in Pakistan, as well as the existing vaccines and their limitations. We also highlight the recent advances in vaccine research and development, such as recombinant and vectored vaccines, thermostable formulations, and novel delivery methods. We conclude by providing some recommendations for future research and development to improve vaccine efficacy, safety, and coverage in Pakistan.

## 2. Epidemiology of PPR in Pakistan

Pakistan is one of the endemic regions where PPR outbreaks have been reported frequently, affecting the livelihoods of millions of smallholder farmers who depend on small ruminants for food security and income generation [1]. The first confirmed case of PPR in Pakistan was reported in 1991 in the Punjab province [25,26]. Since then, PPR has spread to all the provinces and regions of the country, with varying degrees of prevalence and distribution [27]. The prevalence and distribution of PPRV lineages in Pakistan have been investigated by several studies using serological and molecular methods. A national level serological survey conducted in 2014 revealed that the true seroprevalence of PPR in Pakistan was estimated to be 48.5% (95% CI, 46.6–50.3), and 52.9% (95% CI, 50.7–55.1) and 37.7 (95% CI, 34.4–41.0) for goats and sheep, respectively [28]. The sheep and goats exhibited a different seroprevalence pattern, with a higher prevalence in goats. The highest seroprevalence was observed in Balochistan province, followed by Khyber Pakhtunkhwa, Punjab, Sindh, and Gilgit Baltistan. The lowest seroprevalence was recorded in Azad Jammu and Kashmir [29,30,31]. Molecular characterization of PPRV isolates from Pakistan has confirmed that Lineage IV is the predominant and widespread strain, affecting all provinces and regions of the country. Phylogenetic analysis of the fusion gene further reveals that Pakistani PPRV isolates cluster into two distinct subgroups within Lineage IV, designated as IVa and IVb. The IVa subgroup shows a closer genetic relationship to isolates from China, India, and Nepal, while the IVb subgroup is more closely related to isolates from Turkey, Iran, and Iraq [32,33].

**Figure 1:**
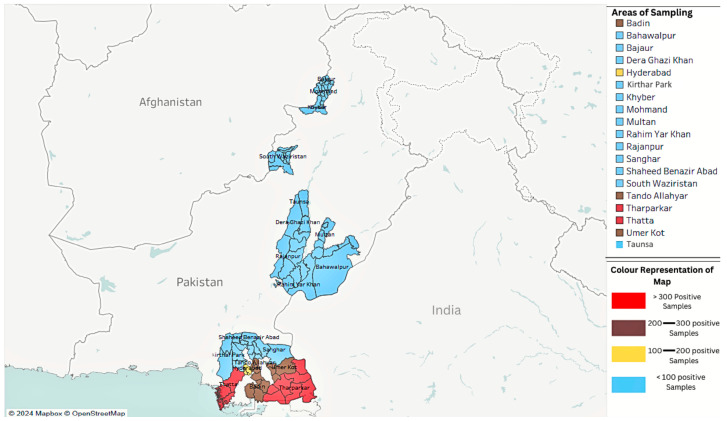
Map showing the distribution of peste des petits ruminants (PPR) positive samples across different areas from 2019 to 2024.

Figure 1 illustrates the provincial distribution of PPR outbreaks in Pakistan, highlighting high-risk areas based on serological and molecular data from national and regional surveys [28,29,30,31].

The risk factors and transmission dynamics of PPR in Pakistan have been studied by various researchers using epidemiological models and surveys [14]. The risk factors associated with PPR infection in small ruminants included age, sex, breed, species, herd size, herd composition, vaccination status, contact with other animals, and movement patterns [31,34,35]. The transmission dynamics of PPR in Pakistan were influenced by the seasonal variations, climatic conditions, and socio-cultural practices. The disease was more prevalent in the winter and spring seasons when the animals were kept in close proximity and shared common grazing and watering sources [34]. The disease was also more prevalent in the regions where the livestock trade and movement were frequent and unregulated, such as the border areas with Afghanistan, Pakistan and Iran [36]. The socio-cultural practices, such as animal sacrifice during religious festivals, animal exchange during marriages, and animal sharing among relatives and neighbors, also facilitated the spread of PPR in Pakistan and other neighbored country [37,38].

**Figure 2:**
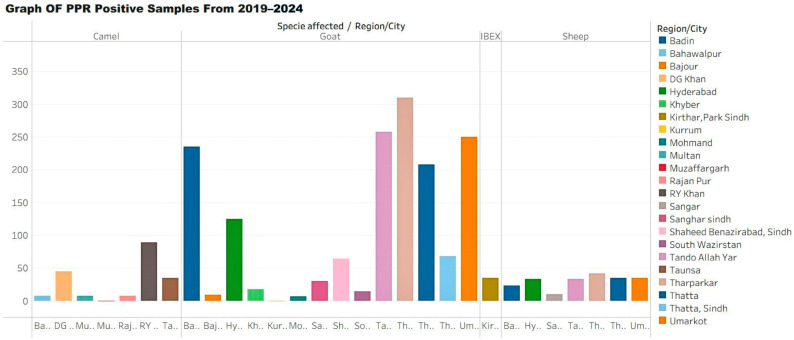
Graph showing the number of peste des petits ruminants (PPR) positive samples by area from 2019 to 2024.

Figure 2 summarizes the transmission dynamics of PPR in Pakistan, incorporating seasonal trends, herd management practices, livestock movement, and socio-cultural factors [34,35,36,37,38].

The impact of PPR on small ruminant production and livelihoods in Pakistan was estimated by several studies using economic and social indicators [38]. The direct economic losses due to PPR in Pakistan were calculated by considering the mortality, morbidity, reduced productivity, and treatment costs of the affected animals. The indirect economic losses due to PPR in Pakistan were calculated by considering the reduced market value, reduced demand, reduced trade, and reduced income of the affected farmers [1]. The social impact of PPR in Pakistan was assessed by considering the food insecurity, poverty, gender inequality, and social stigma of the affected households. The studies showed that PPR caused significant economic and social losses in Pakistan, especially for the poor and marginalized farmers who relied on small ruminants for their livelihoods [39,40]. The surveillance and reporting systems for PPR in Pakistan were evaluated by several studies using the criteria of sensitivity, specificity, timeliness, representativeness, and completeness [14,41,42,43]. The studies revealed that the surveillance and reporting systems for PPR in Pakistan were inadequate and inefficient, due to the lack of resources, infrastructure, coordination, awareness, and incentives [42,44,45]. The passive surveillance system, which relied on the reporting of clinical cases by the farmers and veterinarians, was under-reported and biased, due to the low awareness, low access, and low trust of the stakeholders. The active surveillance system, which involved the collection and testing of samples from the field, was limited and sporadic, due to the lack of funds, equipment, personnel, and protocols. The laboratory diagnosis of PPR in Pakistan was also hampered by the lack of quality, standardization, and validation of the diagnostic methods and kits. The data management and analysis of PPR in Pakistan were also poor and inconsistent, due to the lack of databases, software, and skills [39].

## 3. Diagnosis and Control of PPR in Pakistan

The diagnosis and control of PPR in Pakistan are crucial for the prevention and eradication of the disease, as well as for the protection of small ruminant production and livelihoods [38]. However, the diagnosis and control of PPR in Pakistan face several challenges, such as the lack of resources, infrastructure, coordination, awareness, and incentives. The diagnostic methods for PPR in Pakistan include clinical, serological, and molecular techniques [46,47,48,49]. The clinical diagnosis of PPR is based on the observation of the characteristic signs and lesions of the disease, such as fever, depression, ocular and nasal discharge, erosive stomatitis, pneumonia, diarrhea, and dehydration [28]. However, the clinical diagnosis of PPR is not reliable, as the disease can be confused with other diseases that cause similar symptoms, such as foot and mouth disease, contagious caprine pleuropneumonia, bluetongue, and sheep and goat pox [16,50,51]. The serological diagnosis of PPR is based on the detection of antibodies against PPRV in the serum of the animals, using tests such as competitive enzyme-linked immunosorbent assay (cELISA), immunocapture ELISA (IcELISA), and agar gel immunodiffusion (AGID) [52]. However, the serological diagnosis of PPR has some limitations, such as the inability to differentiate between infected and vaccinated animals (DIVA), the interference with maternal antibodies, and the cross-reactivity with other morbilliviruses [34,53,54]. The molecular diagnosis of PPR is based on the detection of PPRV nucleic acid in the samples of the animals, using techniques such as reverse transcription polymerase chain reaction (RT-PCR), real-time RT-PCR, and loop-mediated isothermal amplification (LAMP) [55]. Recent advancements have expanded molecular diagnostics to include qPCR-based multiplex panels capable of simultaneously detecting PPRV and co-infecting respiratory pathogens, as demonstrated by [56]. Additionally, biomarker- and cytokine-based approaches have emerged as novel supportive diagnostic tools for early-stage PPR detection, as reported by [57]. These modern molecular and immunological tools enhance detection sensitivity, specificity, and early differentiation of infection stages, allowing for genotyping and phylogenetic analysis of circulating PPRV strains. control measures for PPR in Pakistan include biosecurity, quarantine, culling, and vaccination. The biosecurity measures for PPR involve the prevention of contact between infected and susceptible animals, as well as the disinfection of the premises, equipment, and vehicles that may be contaminated with PPRV [38]. The quarantine measures for PPR involve the isolation and observation of the animals that may have been exposed to PPRV, as well as the restriction of their movement and trade [48]. The culling measures for PPR involve the slaughter and disposal of the animals that are confirmed or suspected to be infected with PPRV, as well as the compensation of the owners for their losses. The vaccination measures for PPR involve the administration of vaccines that can induce protective immunity against PPRV in the animals, as well as the monitoring of their efficacy and safety [29]. The national and regional strategies and policies for PPR in Pakistan are aligned with the Global Strategy for the Control and Eradication of PPR, launched by the Food and Agriculture Organization of the United Nations (FAO) and the World Organization for Animal Health (OIE) in 2015, with the goal of eradicating PPR by 2030 [20].

The national strategy for PPR in Pakistan is based on four stages: assessment, control, eradication, and post-eradication [14,41,42]. The assessment stage evaluates epidemiological trends, diagnostic capacity, vaccine availability, and socio-economic impacts, while the control stage emphasizes targeted vaccination, biosecurity enforcement, and outbreak investigation in endemic and high-risk zones such as the border areas of Khyber Pakhtunkhwa and Baluchistan. Currently, Pakistan is implementing the Government of Pakistan, Ministry of National Food Security & Research, National Peste des Petits Ruminants (PPR) Eradication Programme; Phase-I, Risk-Based PPR Control in Sheep and Goats of Pakistan (2021–2025)**.** This national program focuses on risk-based vaccination, community awareness, and strengthened passive surveillance. To date, it has vaccinated over 70 million small ruminants, achieving a significant reduction in outbreak frequency in targeted districts. The eradication phase will focus on verifying disease-free zones, while the post-eradication phase emphasizes sustained surveillance and prevention of viral re-entry from neighboring endemic countries [58].

The regional strategy for PPR in Pakistan involves the coordination and collaboration with the neighboring countries and regions, such as Afghanistan, Iran, India, and China, where PPR is also endemic or epidemic, to harmonize the surveillance, diagnosis, and control of PPR across the borders [36,59].

## 4. Pakistan’s National PPR Control Program

Pakistan’s strategic efforts to combat Peste des Petits Ruminants (PPR) are framed within its national livestock policies and its commitment to the WOAH/FAO Global Eradication Strategy. According to recent assessments, Pakistan is currently positioned in Stage 2 (Risk Reduction) of the Progressive Control Pathway (PCP) for PPR [60]. In this stage, the national objective is to reduce infection pressure in targeted populations and geographic areas through the implementation of risk-based, controlled vaccination programs, rather than achieving nationwide freedom from the disease. The foundation of this control program is the delineation of risk zones, which enables the efficient allocation of limited resources. Pakistani authorities classify areas as high-risk based on multiple criteria, including a history of recurrent outbreaks, proximity to borders with endemic countries such as Afghanistan and Iran, and the density of small ruminant populations along trade and migration routes [38,61]. This risk mapping approach ensures that interventions are focused where they are most needed, particularly in the border regions of Khyber Pakhtunkhwa and Balochistan, which serve as key livestock movement corridors. Within these defined high-risk zones, the primary control measure involves targeted vaccination campaigns. The strategy includes the prophylactic use of the live-attenuated Nigeria 75/1 vaccine, often produced domestically at the Veterinary Research Institute (VRI) Lahore, to build herd immunity and reduce virus circulation [62,63]. This vaccination strategy is complemented by passive and active surveillance systems designed to ensure early outbreak detection. When a PPR outbreak is confirmed, immediate response measures are triggered, including quarantine enforcement, restriction of animal movement, and ring vaccination around the epicenter to contain viral spread. Despite the clear policy framework and national coordination, implementation challenges persist. These include logistical difficulties in vaccinating nomadic and transhumant herders, limited cold chain infrastructure in remote districts, inadequate funding for sustained vaccination coverage, and ongoing virus incursions across porous borders. Bridging these operational gaps is vital for Pakistan to transition from Stage 2 (Risk Reduction) to Stage 3 (Control), demonstrating tangible progress toward the national and global goal of PPR eradication. The Government of Pakistan, through the Ministry of National Food Security and Research (MNFSR), is leading these efforts under the National PPR Eradication Programme: Phase I (2021–2025), which is focused on risk-based control, surveillance enhancement, and regional coordination with neighboring countries. Successful transition to the next PCP stage will depend on sustained vaccination coverage, active sero-monitoring, and strengthened cross-border collaboration, aligning national actions with the global PPR eradication goal by 2030.

## 5. PPR Vaccination Strategies and Progress in Pakistan

In Pakistan, vaccination against Peste des Petits Ruminants (PPR) serves as the cornerstone of disease prevention and control under the National PPR Eradication Programme (Phase I, 2021–2025), which is fully funded and implemented by the Government of Pakistan through the Ministry of National Food Security and Research (MNFSR). The program is coordinated by the Animal Husbandry Commissioner’s Office, with technical guidance and strategic alignment provided by the Food and Agriculture Organization (FAO) and the World Organisation for Animal Health (WOAH, formerly OIE).The country primarily employs a live-attenuated vaccine derived from the Nigeria 75/1 strain, locally produced and quality-assured by the Veterinary Research Institute (VRI), Lahore, under the supervision of the Animal Husbandry Commissioner’s Office [64]. In addition, the Sindh Poultry Vaccine Centre (SPVC), Tandojam, contributes to local vaccine production and regional distribution within Sindh province. These vaccines are used in localized mass vaccination campaigns, particularly targeting high-risk districts in Sindh, Punjab, Balochistan, and Khyber Pakhtunkhwa, where seropositivity and outbreak recurrence remain consistently high [40,65,66]. Despite the availability of effective vaccines, national immunization coverage remains suboptimal, averaging only 35–45% of the estimated 120 million small-ruminant population [62]. Coverage gaps arise due to cold-chain fragility, limited logistical infrastructure, funding constraints, and low farmer compliance and awareness, particularly in semi-arid and tribal regions where pastoral mobility complicates systematic vaccination [67,68]. These weaknesses lead to uneven herd immunity and periodic re-emergence of PPR outbreaks, even with ongoing campaigns. To address vaccine thermolability, national research efforts have prioritized thermostabilization and formulation improvements. Locally tested thermostable preparations of the Nigeria 75/1 vaccine have shown full protective efficacy after exposure to 37 °C for up to 14 days under Sindh’s semi-arid conditions [68]. Furthermore, recent research [69] demonstrated that lentinan, a β-glucan-based natural adjuvant, significantly enhanced PPR vaccine immunogenicity by increasing both antibody titers and cytokine-mediated immune responses [69]. These findings support the integration of novel adjuvant systems into national vaccine formulations to enhance immune durability, memory response, and field efficacy under challenging environmental conditions. Parallel efforts at the National Veterinary Laboratory (NVL), Islamabad, are focused on molecular characterization and phylogenetic mapping of circulating lineage IV strains of the PPR virus to ensure homologous antigenic matching and continuous genomic surveillance for emerging mutations [65,66]. Collectively, these developments underscore Pakistan’s advancing technical capacity and institutional commitment toward achieving the FAO–WOAH Global Eradication Goal for PPR by 2030 [60]. Nevertheless, large-scale field validation, post-vaccination seromonitoring, and farmer-focused awareness programs remain vital to sustaining vaccine-driven herd immunity and preventing resurgence in endemic clusters.

## 6. Comparative Aspects of PPR Vaccination in Sheep and Goats

A one-size-fits-all vaccination approach for small ruminants is suboptimal due to marked interspecies differences. Firstly, goats are profoundly more susceptible to PPR, suffering mortality rates that can surpass 80% in naïve herds, compared to 10–40% in sheep [61]. Secondly, their immune responses diverge: sheep mount a consistently robust and long-lasting antibody response to vaccines like Nigeria 75/1, while goats often show variable and sometimes weaker neutralizing antibody titers, with evidence pointing to a greater reliance on cell-mediated immunity and potentially shorter duration of protection [11]. These biological disparities translate directly to field practice. The heightened susceptibility of goats mandates their prioritization during outbreak containment [70]. Furthermore, their quicker waning immunity suggests they may need more frequent booster vaccinations and should be the primary focus of post-vaccination seromonitoring campaigns

## 7. Recent Advances in Vaccine Research and Development

The global endeavor to develop next-generation PPR vaccines is largely driven by the inherent limitations of existing Live Attenuated Vaccines (LAVs), including their stringent cold-chain requirements, the inability to differentiate infected from vaccinated animals (DIVA), and potential safety concerns in immunocompromised or pregnant animals [64]. For a country like Pakistan, where high ambient temperatures and an unreliable cold chain are pervasive issues, these limitations are not merely theoretical but represent critical operational failures [68]. Consequently, the pursuit of thermostable, recombinant, and DIVA-compatible vaccines has become a paramount national research priority.

The direction of this research is critically guided by precise local epidemiological data. Nationwide phylogenetic studies have consistently shown that the PPR virus strains circulating in Pakistan are not random but belong almost exclusively (>98%) to lineage IV, with further subdivisions into genetically distinct sub-clusters IVa and IVb that show phylogenetic links to viruses from China and the Middle East, respectively [11]. This finding is crucial because it underscores the necessity for antigenic matching to ensure that vaccines deployed in Pakistan elicit the strongest possible immune response against the viruses actually present in the field.

In response, Pakistan’s national research capacity is being actively mobilized. Leading institutions, including the University of Veterinary and Animal Sciences (UVAS) in Lahore and the National Veterinary Laboratories (NVL) in Islamabad, are at the forefront of developing second-generation vaccines tailored to the local context [60,71]. Their work leverages cutting-edge platforms. Recombinant and vectored vaccines, which use benign viruses as carriers for PPRV genes, are a major focus as they inherently allow for DIVA strategies. Promisingly, preliminary challenge studies for a UVAS-developed recombinant candidate vaccine demonstrated a 100% protection rate in goats exposed [65,72]. Furthermore, global and local experimental data confirm that recombinant vaccines expressing the Hemagglutinin (H) or Fusion (F) proteins from lineage IV viruses can induce neutralizing antibody titers that are 4 to 8 times higher than those generated by the conventional Nigeria 75/1 (lineage II) vaccine [73].

The most advanced technological approach being explored is reverse genetics, a technique that allows for the precise engineering of the entire PPRV genome. This platform enables scientists to create tailored vaccine strains for instance, by building upon the proven Nigeria 75/1 backbone but inserting key immunogenic genes from the local lineage IV viruses thereby designing vaccines that are both safe and optimally effective against the circulating Pakistani strains.

Pakistan is transitioning from being a mere consumer of global vaccine technology to an active participant in its innovation. The collaboration between its leading academic and diagnostic institutions is yielding promising, locally relevant vaccine candidates. The critical next steps involve channeling investment and effort into large-scale field efficacy trials, rigorous safety profiling, and navigating the regulatory pathways to integrate these advanced vaccine prototypes into the NPCEP, ultimately accelerating the country’s progress towards eradication. A summary of recent global and regional advancements in PPR vaccine research, including Pakistan’s contributions, is presented in Table 1.

## 8. Prospects and Challenges for the Commercial Production of PPR Vaccines

The transition of a promising vaccine candidate from the laboratory to a commercially available product is a complex and costly process. Although the pipeline of next-generation PPR vaccines including recombinant, vectored, and DIVA compatible candidates is robust, their path to market is non-trivial [79]. Significant hurdles in scale-up and cost-effectiveness remain. Producing vaccines under stringent Good Manufacturing Practice (GMP) conditions requires substantial investment in infrastructure and quality control, which can be prohibitive for manufacturers targeting affordable products for developing economies [80]. This challenge is underscored by a dynamic intellectual property landscape, with numerous patents protecting various platforms, from improved live-attenuated strains to novel recombinant technologies, which must be navigated. For countries like Pakistan, this global challenge presents a specific opportunity. The well-established local production of the Nigeria 75/1 vaccine at institutions such as the Veterinary Research Institute (VRI) in Lahore provides a critical foundational platform. This existing capacity could be strategically leveraged for technology transfer agreements to produce advanced thermostable formulations, thereby reducing reliance on international suppliers and ensuring a stable, cost-effective vaccine supply for national and regional eradication campaigns [81]. Ultimately, fostering strong public–private partnerships is essential to de-risk investment for pharmaceutical companies, manage intellectual property, and ensure these advanced vaccines reach the farmers who need them most.

## 9. Thermostable Formulations

Thermostable PPR vaccines represent a major breakthrough in the control and eradication of Peste des Petits Ruminants, especially in developing countries such as Pakistan, where climatic extremes and logistical barriers frequently undermine the cold-chain integrity required for conventional vaccines. These vaccines are developed by incorporating stabilizing agents such as trehalose and gelatin, which enhance thermal stability, extend shelf-life, and minimize dependence on refrigeration during transportation and storage [82,83]. This advancement is of particular significance for Pakistan, where rugged geography, high ambient temperatures, and weak infrastructure often restrict access to remote pastoral areas. By simplifying field vaccination and improving outreach, thermostable formulations offer a practical and sustainable solution for protecting small ruminant populations in isolated or nomadic settings [84,85,86]. Several experimental formulations employing various stabilizers have shown promising results under laboratory and field conditions [87,88]. Among these, the thermostable live-attenuated Nigeria 75/1 vaccine is a landmark innovation, as it meets FAO/WOAH performance standards by retaining potency for more than three days at 45 °C [89,90]. Field trials conducted under Pakistan’s semi-arid conditions confirmed its high safety, immunogenicity, and efficacy, reinforcing its potential as a core component of the National PPR Control Programme, particularly for hard-to-reach districts in Balochistan and Khyber Pakhtunkhwa [83]. Comparable advancements have also been achieved in neighboring India, where the Sungri 96 thermostable vaccine demonstrated similar success, further validating the regional feasibility and reliability of thermostable vaccine technology [19,91]. Moreover, alternative stabilizers such as mannitol, sorbitol, and polymer-based matrices have been explored, each showing favorable results in enhancing vaccine durability and field stability [92]. The strategic incorporation of thermostable vaccine technology into Pakistan’s national immunization framework is therefore crucial for overcoming logistical bottlenecks, ensuring consistent vaccine performance, and achieving equitable and sustained PPR control across all ecological zones of the country.

## 10. Novel Delivery Methods

The novel delivery methods for PPR vaccines are based on the administration of vaccines through alternative routes or devices, which can bypass the conventional needle-based injection. The advantages of novel delivery methods include the reduction in pain, fear, and anxiety, the improvement of compliance and acceptability, and the enhancement of immunogenicity and protection [93]. For Pakistan, where mass vaccination campaigns are logistically complex and farmer compliance can be variable, such methods could significantly increase participation rates and campaign efficiency. Several novel delivery methods for PPR vaccines are being developed, including oral, intranasal, aerosol, and needle-free approaches [94,95]. This approach would be particularly advantageous for reaching nomadic or free-grazing herds in Pakistan’s extensive rangeland systems. However, oral delivery of PPR vaccines faces some challenges, such as the degradation of the vaccines by the gastric acid and enzymes, the low absorption of the vaccines by the intestinal epithelium, and the induction of immunological tolerance or anergy [96,97]. Oral delivery enables vaccine ingestion to induce systemic and mucosal immunity, though it faces challenges like gastric degradation and potential immune tolerance. Similarly, intranasal administration through the nasal cavity can stimulate both systemic and mucosal immune responses, but encounters obstacles such as mucociliary clearance, low nasal epithelium permeability, and possible immunological tolerance [95]. Aerosol delivery of PPR vaccines involves the inhalation of vaccines by the animals, which can induce both systemic and mucosal immune responses, as well as pulmonary tolerance [22,98,99,100]. This method could enable rapid mass vaccination in the dense livestock markets and communal holding areas common in Pakistan. However, aerosol delivery of PPR vaccines also faces some challenges, such as the deposition of the vaccines in the upper airways, the clearance of the vaccines by the alveolar macrophages, and the induction of immunological tolerance or anergy [101]. Needle-free delivery of PPR vaccines administers vaccines through the skin or mucosa using devices like jet injectors, microneedles, or electroporation, inducing systemic and mucosal immune responses by targeting antigen-presenting cells and lymphoid tissues [102,103,104]. The use of needle-free devices could improve safety and speed during large-scale vaccination drives in Pakistan, reducing the need for highly trained personnel and addressing needle-phobia among handlers. However, needle-free delivery of PPR vaccines also faces some challenges, such as the pain, bleeding, and infection associated with some devices, the variability of the skin or mucosa thickness and permeability, and the optimization of the vaccine dose and formulation. A concerted effort to pilot and evaluate the most suitable novel delivery methods within Pakistan’s specific infrastructure is a vital next step for enhancing the national PPR control program.

## 11. Intellectual Property Landscape

The global PPR vaccine patent landscape reflects key technological advances, spanning from conventional live-attenuated vaccines to next-generation thermostable, DIVA-compatible, and DNA-based platforms [105,106]. This progression underscores a committed international effort to develop practical solutions for PPR eradication [107].

## 12. Current and Potential Strategies for Vaccine Deployment

The vaccine deployment for PPR involves the planning, implementation, and evaluation of the vaccination activities, which aim to achieve the optimal coverage and impact of the vaccines in the target populations [52]. The vaccine deployment for PPR depends on several factors, such as the availability, accessibility, affordability, acceptability, and quality of the vaccines, as well as the logistics, infrastructure, coordination, communication, and monitoring of the vaccination programs [23]. In Pakistan, these factors are profoundly influenced by the country’s diverse husbandry systems, from intensive farms in Punjab to the vast nomadic and transhumant herds in Balochistan and Khyber Pakhtunkhwa, necessitating a flexible and multi-pronged deployment strategy. The current strategies for vaccine deployment for PPR include mass vaccination, targeted vaccination, and ring vaccination. Mass vaccination involves the vaccination of all susceptible animals in a defined area or region, regardless of their exposure or infection status [108]. Mass vaccination is suitable for the areas where PPR is endemic or epidemic, and where the resources and infrastructure are sufficient to reach a high coverage (>80%) of the target population. Mass vaccination can rapidly reduce the disease incidence and transmission and create herd immunity [109]. This approach may be most feasible in Pakistan’s central agricultural districts with higher veterinary service density. However, mass vaccination also has some drawbacks, such as the high cost, the waste of vaccines, the interference with surveillance and eradication, and the potential adverse effects. Targeted vaccination involves the vaccination of specific subgroups of animals that are at higher risk of exposure or infection, such as young animals, pregnant animals, or animals in certain locations or seasons [110]. Targeted vaccination is suitable for the areas where PPR is sporadic or emerging, and where the resources and infrastructure are limited to achieve a high coverage of the whole population [111]. Targeted vaccination can efficiently protect the most vulnerable animals and reduce the disease morbidity and mortality. For Pakistan, targeting key entry points like border regions with Afghanistan and Iran, or major livestock markets, could be a highly strategic use of resources. However, targeted vaccination also has some limitations, such as the difficulty of identifying and reaching the target subgroups, the low impact on disease transmission and elimination, and the potential selection of resistant strains. Ring vaccination involves the vaccination of the animals that are in contact or proximity with the confirmed or suspected cases of PPR, as well as the animals in the surrounding areas or regions [112]. Ring vaccination is suitable for the areas where PPR is rare or absent, and where the surveillance and diagnosis are rapid and accurate to detect and contain the disease outbreaks. The effectiveness of ring vaccination in Pakistan is currently hampered by delays in disease reporting and diagnostic confirmation, highlighting a critical area for system strengthening. Ring vaccination can effectively prevent the spread of the disease and eliminate the residual infection. However, ring vaccination also has some challenges, such as the complexity of tracing and vaccinating the contacts, the delay of the vaccination response, and the dependence on the cooperation and compliance of the stakeholders. The potential strategies for vaccine deployment for PPR include novel approaches that can improve the efficiency, effectiveness, and sustainability of the vaccination programs [113]. For example, the use of thermostable formulations can eliminate or reduce the need for cold chain and facilitate the vaccine delivery and administration in remote and rural areas. As previously discussed, the adoption of thermostable vaccines is a paramount strategy for Pakistan to overcome its specific logistical constraints. The use of novel delivery methods can reduce the pain, fear, and anxiety associated with needle-based injection, and improve the compliance and acceptability of the vaccination [114]. Piloting novel delivery methods like oral or needle-free systems could be transformative for increasing farmer participation in Pakistan’s diverse cultural and farming contexts. The use of recombinant and vectored vaccines can enable the differentiation between infected and vaccinated animals (DIVA) and enhance the surveillance and monitoring of the vaccination impact. Implementing a DIVA strategy in the long term would be invaluable for Pakistan to accurately assess progress toward eradication. The use of simulation models and decision support tools can optimize the vaccination strategies and policies, based on the epidemiological, economic, and social parameters. Developing such models with Pakistan-specific data on livestock mobility, population density, and resource allocation is an essential research priority to guide the national eradication campaign.

Table 2 provides a consolidated summary of major serological and molecular studies reporting PPR prevalence, sample sizes, and lineage distributions across Pakistani provinces. The table is introduced here to allow direct comparison of prevalence between goats and sheep and among various regions, contextualizing the epidemiological overview provided in this subsection.

## 13. Conclusions and Future Perspectives

The global eradication of Peste des Petits Ruminants (PPR) by 2030 is an ambitious goal that demands a decisive shift from conventional control to a strategic, technology-driven eradication campaign. The foundation of this effort lies in the widespread adoption of next-generation vaccines, specifically thermostable formulations that bypass cold-chain limitations and DIVA-compatible (Differentiating Infected from Vaccinated Animals) systems that are essential for accurate surveillance and proving freedom from disease. For Pakistan, this global initiative presents a critical opportunity to translate its existing infrastructure into regional leadership. By leveraging its domestic vaccine production capabilities at the Veterinary Research Institute (VRI) and aligning its national control program with international standards, Pakistan can serve as a model for effective PPR management. Prioritizing the integration of advanced vaccines into its national strategy, bolstered by robust surveillance and data-driven deployment, will be crucial for progress. Ultimately, success hinges on a unified approach that seamlessly connects scientific innovation with on-the-ground implementation. This requires sustained political commitment, increased investment in local research, and strengthened cross-border collaboration. By championing this integrated strategy, Pakistan can not only secure the livelihoods of its millions of small ruminant farmers but also cement its legacy as a key contributor to one of the most significant veterinary public health achievements of our time.

## Figures and Tables

**Table 1:** Summary of Recent Advancements and Key Characteristics of Peste des Petits Ruminants (PPR) Vaccine Candidates and Platforms across Different Regions.

Country/Region	Vaccine Type	Target Species	Key Outcomes	Efficacy/Results	Limitations and Challenges	Reference
Nigeria/Africa	Live-attenuated (Nigeria 75/1)	Sheep and Goats	Cross-protection across all lineages; long-lasting immunity (>3 years); foundation for global eradication	>95% protection in challenge studies	Strict cold-chain requirement; not DIVA-compatible; thermolabile	[74]
India	Recombinant capripox–PPR chimera	Goats	Dual protection against PPR and goat-pox; DIVA capability; improved thermal stability	92% seroconversion; 100% protection from challenge	Pre-existing immunity to vector may reduce efficacy; complex manufacturing	[75]
Pakistan	Thermostable live-attenuated Nigeria 75/1	Sheep and Goats	Stable at 45 °C for >3 days; maintained immunogenicity under field conditions; ideal for remote areas	95% seroconversion in field trials	Limited commercial availability; higher production cost	[68]
Pakistan	Recombinant vaccine candidates (H and F genes of lineage IV)	Goats	Strong neutralizing antibody response; protection against local lineage IV strains; potential for DIVA strategies	4–8× higher antibody titers than conventional vaccines	Requires extensive field validation; regulatory approval pending	[76]
Global review	DIVA vaccine platforms	Sheep and Goats	Enables serosurveillance during vaccination; critical for eradication programs	High immunogenicity (varies by platform)	Not yet commercially available; higher cost; requires companion diagnostic tests	[77]
China	Vectored DIVA vaccine	Goats	Differentiates infected from vaccinated animals; excellent safety profile; strong immune response	94% protection in challenge studies	Limited field data; regulatory approval required; scalability uncertain	[78]

**Table 2:** Diagnostic results of peste des petits ruminants (PPR) from 2019 to 2024, indicating region/city, species affected, number of samples tested, positive cases, diagnostic methods, and references.

Region/City	Specie Affected	Total Number of Sample	No. of Positive Sample	Diagnostic Test	Reference
Sindh	Goat	4900	1386	Competitivc-ELISA	[49]
	Sheep	800	201		
Hyderabad	Goat	605	125		
	Sheep	260	33		
Thatta	Goat	835	208		
	Sheep	110	35		
Umarkot	Goat	905	250		
	Sheep	90	35		
Badin	Goat	780	235		
	Sheep	135	23		
Tando Allah Yar	Goat	865	258		
	Sheep	120	33		
Tharparkar	Goat	910	310		
	Sheep	85	42		
Sanghar Sindh,	Goat	100	30	Ic-ELISA.	[114]
	Sheep	100	10		
Shaheed Benazirabad, Sindh	Goat	100	64	(c-ELISA)	[115]
Sakrand		25	18		
Qazi Ahmad		25	18		
Nwabshah		25	16		
Daur		25	12		
Thatta, Sindh	Goat	100	68	(c-ELISA)	[116]
Jhampir		25	21		
Jhirk		25	18		
Gharo		25	16		
Mirpursakro		25	13		
Border Area of Pakistan with Afghanistan	Goat	308	49	(c-ELISA)	[117]
Khyber		80	18		
Kurrum		80	0		
Bajour		50	09		
Mohmand		50	07		
South Wazirstan		48	15		
Southern Punjab	Camel	992	193	(c-ELISA) and (RT-PCR)	[118]
Bahawalpur		30	8		
DG Khan		207	45		
Multan		100	8		
Muzaffargarh		17			
Rajan Pur		152	8		
RY Khan		254	89		
Taunsa		232	35		
Sindh, Pakistan	Camel	200	17	(c-ELISA)	[27]
UmarKot		90	5		
Tharparkar		110	12		
Kirthar, Park Sindh	Ibex		35		[119]

## Data Availability

Data are contained within the article.
